# Circulating complement component 4d (C4d) correlates with tumor volume, chemotherapeutic response and survival in patients with malignant pleural mesothelioma

**DOI:** 10.1038/s41598-017-16551-7

**Published:** 2017-11-28

**Authors:** Thomas Klikovits, Paul Stockhammer, Viktoria Laszlo, Yawen Dong, Mir Alireza Hoda, Bahil Ghanim, Isabelle Opitz, Thomas Frauenfelder, Thi Dan Linh Nguyen-Kim, Walter Weder, Walter Berger, Michael Grusch, Clemens Aigner, Walter Klepetko, Balazs Dome, Ferenc Renyi-Vamos, Rudolf Oehler, Balazs Hegedus

**Affiliations:** 1 Translational Thoracic Oncology Laboratory, Division of Thoracic Surgery, Department of Surgery, Comprehensive Cancer Center Vienna, Medical University of Vienna, Waehringer Guertel 18–20, 1090 Vienna, Austria; 2 Department of Biomedical Imaging and Image-guided Therapy, Division of Molecular and Gender Imaging, Medical University of Vienna, Waehringer Guertel 18–20, 1090 Vienna, Austria; 3 Division of Thoracic Surgery, University Hospital Zurich, Raemistrasse 100, 8091 Zurich, Switzerland; 4 Department of Diagnostic and Interventional Radiology, University Hospital Zurich, Raemistrasse 100, 8091 Zurich, Switzerland; 5 Institute of Cancer Research, Comprehensive Cancer Center Vienna, Medical University of Vienna, Borschkegasse 8a, 1090 Vienna, Austria; 6 Department of Thoracic Surgery, Ruhrlandklinik, University Hospital Essen, Tueschener Weg 40, 45239 Essen, Germany; 7 National Koranyi Institute of Pulmonology, Piheno út 1, 1121 Budapest, Hungary; 8 Department of Thoracic Surgery, National Institute of Oncology and Semmelweis University, Üllői út 26, 1085 Budapest, Hungary; 9 Department of Surgery, Comprehensive Cancer Center Vienna, Medical University of Vienna, Waehringer Guertel 18–20, 1090 Vienna, Austria; 10 MTA-SE Molecular Oncology Research Group, Hungarian Academy of Sciences – Semmelweis University, Üllői út 26, 1085 Budapest, Hungary

## Abstract

Only limited information is available on the role of complement activation in malignant pleural mesothelioma (MPM). Thus, we investigated the circulating and tissue levels of the complement component 4d (C4d) in MPM. Plasma samples from 55 MPM patients, 21 healthy volunteers (HV) and 14 patients with non-malignant pleural diseases (NMPD) were measured by ELISA for C4d levels. Tissue specimens from 32 patients were analyzed by C4d immunohistochemistry. Tumor volumetry was measured in 20 patients. We found no C4d labeling on tumor cells, but on ectopic lymphoid structures within the tumor stroma. Plasma C4d levels did not significantly differ between MPM, HV or NMPD. Late-stage MPM patients had higher plasma C4d levels compared to early-stage (p = 0.079). High circulating C4d was associated with a higher tumor volume (p = 0.047). Plasma C4d levels following induction chemotherapy were significantly higher in patients with stable/progressive disease compared to those with partial/major response (p = 0.005). Strikingly, patients with low C4d levels at diagnosis had a significantly better overall survival, confirmed in a multivariate cox regression model (hazard ratio 0.263, p = 0.01). Our findings suggest that circulating plasma C4d is a promising new prognostic biomarker in patients with MPM and, moreover, helps to select patients for surgery following induction chemotherapy.

## Introduction

Malignant pleural mesothelioma (MPM) is a devastating malignancy with a strong link to prior asbestos exposure^1–3^. With a median overall survival (OS) of 9 to 12 months following diagnosis, MPM remains associated with a fatal prognosis. Due to the rising incidence, accurate diagnosis and management of MPM are urgently needed^4,5^. Particularly, biomarkers that may facilitate the diagnostic process or indicate susceptibility to specific treatment are of utmost interest in order to improve survival.

The complement cascade is a major component of the innate immunity and it essentially contributes to immune surveillance, cell homeostasis and tissue regeneration^6,7^. However, there is mounting evidence that the complement cascade is also involved in cancer development and progression^6,8–11^. One complement-related protein is the degradation protein C4d, a stable cleavage product of C4, accumulating following classical and lectin pathway activation^12–14^. C4d deposition in a tissue is regarded as an indirect proof of an activation of the complement cascade^13^. C4d is usually bound stably to the target structure but may eventually enter the circulation. Of note, C4d is routinely used as a tissue biomarker to investigate allograft tissue rejections in kidney transplants^13–15^. C4d has already been found to have a prognostic role in different types of cancer^12,13,16^. Ajona *et al*. found increased C4d levels both in plasma and bronchoalveolar lavage (BAL) samples from lung adenocarcinoma patients. Patients with elevated circulating as well as tumor tissue C4d levels had worse prognosis^12,16^. Furthermore, C4d expression has been shown to be associated with disease stage or poor prognosis in different other tumor types including astrocytoma and oropharyngeal squamous cell cancer^13,17^.

There is limited information about the role of the complement system in asbestos-exposed individuals and MPM development. In 2002, Galani *et al*. investigated a cohort of patients with or without pleural calcifications (PCs) due to prior asbestos exposure. C4 was only present in BAL samples of patients with PCs but not in those without PCs. Subjects with PCs were assumed to develop MPM less frequently^18^. Zerva *et al*. investigated several factors associated with humoral immunity in patients environmentally exposed to asbestos. Those patients had significantly increased serum C3 levels independently of the presence of PCs when compared to healthy controls. Although C3 is the target protein of activated C4, serum C4 levels did not differ between these groups^19^. Unfortunately the authors did not provide any data on the on the activation marker C4d. By analyzing mesothelioma pleural effusions, Nabil *et al*. demonstrated that complement factor H, a restrictive cofactor for the cleavage of C3b, potentially recruits monocytes and granulocytes to the malignant site via chemotactic function and thus supports malignant cell phagocytosis^20^.

Since the complement degradation product C4d has been shown to have a strong prognostic relevance particularly in lung adenocarcinoma, we investigated the tissue and circulating levels of C4d in MPM patients and compared these data with tumor load, chemotherapy response and clinicopathological parameters. Finally, we assessed the potential prognostic relevance of circulating C4d levels in patients with MPM.

## Patients and Methods

### Patients

Clinical data and plasma samples of 55 consecutive, histologically verified MPM patients were collected at the time of diagnosis (n = 30) or before curative intent surgery after induction chemotherapy (n = 25) at the Medical University of Vienna between May 2011 and December 2014. In detail, all patient blood and tissue samples were retrieved before any diagnostic or curative intent surgical intervention or in case of post-chemotherapy samples before surgical resection. In consequence, acute clinical infections were ruled out at the time of sample collection. In 12 patients, plasma samples at the time of diagnosis as well as before surgery were available, representing pre- and post-chemotherapy samples. Of 32 of these 55 patients, formalin fixed and paraffin embedded (FFPE) tissue specimens were collected for immunohistochemistry analyses. Furthermore, plasma samples from an age-matched cohort of 21 healthy volunteers as well as from 14 patients diagnosed with non-malignant pleural diseases were included. Individual written informed consent was obtained from each individual and the study was performed after approval of the Ethics Committee at the Medical University of Vienna (#904/2009). Patients gave written informed consent to the inclusion of the material pertaining to them, that they acknowledge that they cannot be identified via the paper; and that they are fully anonymized. All methods included in this manuscript were performed in accordance with the relevant guidelines and regulations.

### Health and safety during experiments

All experiments that were reported in this manuscript were done complying with all mandatory laboratory health and safety procedures.

### Collection of blood

Blood was collected via peripheral venous puncture from MPM patients and controls into 10 ml EDTA Vacutainers (BD Biosciences). Within 30 min after blood collection centrifugation was performed. Plasma supernatant was stored in aliquots at −80 °C until use.

### ELISA assay

C4d and C3a fragments enzyme-linked immunosorbent assay (ELISA) kits were purchased from Quidel (San Diego, CA, USA). For the analyses of C4d and C3a, samples were diluted 1:70 and 1:200, respectively, according to the manufacturers’ instructions. Standard curve generations and measurements in duplicates were performed according to the guidelines of the company.

### Immunohistochemistry

To obtain information about C4d presence within the tumor tissue and to compare these tissue levels with circulating C4d levels, 32 FFPE MPM tissue specimens were collected and analyzed by C4d immunohistochemical staining. Additionally, we performed immunohistochemical staining for C1q in 14 FFPE tissue specimens deriving from patients with high (n = 7) and low (n = 7) circulating C4d levels. After the sections were deparaffinized and rehydrated, endogenous peroxidase activity was blocked with 0.3% H2O2 in phosphate buffered saline. Heat-induced epitope retrieval was performed by using TRIS/EDTA buffer (pH = 9). Primary antibodies (anti-human C4d; Biomedica, Vienna, Austria and anti-human C1q; ab71089, Abcam, Cambridge, UK) were diluted 1:500 and 1:1000, respectively, and incubated for 60 min at RT. Antibody binding was detected with the UltraVision LP Detection System (Lab Vision Corporation, Fremont, CA, USA). Color development was achieved by DAB followed by hematoxylin counterstaining. Stainings were evaluated by a pathologist.

### Tumor volumetry

In 20 of 55 patients, tumor volumetric measurement was available. Volumetric analyses were performed in patients with digitally available CT imaging data before and/or following chemotherapy. Chest CT images were analyzed by using dedicated software featuring semi-automatic segmentation with linear interpolation, allowing manual adjustments if necessary (Myrian; Intrasense, Paris, France) - as previously described^21^.

In summary, the segmentation and tumor volume quantification contained the following steps: the normal lung tissue, including bronchi and vessels, was marked semi-automatically by thresholding and region growing. Then pleural effusion and atelectatic lung areas were marked with a magnetic lasso function. After fixing normal lung tissue, pleural effusion, atelectatic lung and the outer part of the pleura was segmented semi-automatically. By using a lineal algorithm, interpolation between the marked slices was applied automatically. Finally, tumor volume was calculated by multiplying the sum of the voxels. Data was independently analyzed by two radiologists.

### Cut-off evaluation

To investigate the role of circulating plasma levels of C4d, first we stratified our cohort by the cut-off of 3 µg/mL which was previously used in lung adenocarcinoma^12^. Nevertheless, because only one patient had C4d plasma levels above 3 µg/mL at time of diagnosis, this cut-off value proved to be not suitable for MPM. However, when we analyzed circulating C4d values of patients with early- and late-stage disease, we found a distinct separation when using 1.5 µg/mL concentration as a potential cut-off. We also found that 1.5 µg/mL level was the best cut-off in our cohort by performing ROC curve analysis and Youden’s Index calculation for twelve months’ survival (data not shown). Hence, calculations described below were performed by using 1.5 µg/mL as cut-off.

### Statistical analysis

Categorical data was compared by performing Fishers’ exact or chi-square tests. Statistical differences between two groups were tested by Mann-Whitney U test. The significance of potential correlations of continuous parameters was investigated by using Pearson correlation. Overall survival (OS) was defined as time between initial MPM diagnosis and date of death or last follow-up. OS was estimated by the Kaplan-Meier method and a log rank test was used to calculate survival differences between two groups. A multivariate cox regression model was used to calculate hazard ratios and 95% confidence intervals for factors independently influencing OS. To investigate the accuracy of C4d as a predictor for survival and to distinguish between MPM and non-malignant pleural disease, Receiver Operating Characteristics (ROC) curve analysis was used. For each cut-off point, the Youden’s Index was calculated (sensitivity + specificity). All results were considered statistically significant when p < 0.05 two-sided. Analyses were performed using the SPSS Statistics 23.0 package (SPSS Inc., Chicago, IL, USA) and GraphPad Prism 5.0.

## Results

### Clinicopathological characteristics of the study cohort

The clinicopathological parameters of the patient cohort are presented in Table 1. A total of 34 patients (61.8%) underwent multimodality treatment, consisting of induction chemotherapy (platinum-based) followed by macroscopic radical resection with or without adjuvant intensity-modulated radiation therapy (IMRT). In 31 (91.2%) of these patients, extrapleural pneumonectomy (EPP) was performed. Neo-adjuvant chemotherapy was applied in 35 patients (63.6%) and consisted of three to four cycles of platinum-based chemotherapy. Fifteen patients (38.2%) received chemotherapy and/or radiation therapy only due to advanced disease at the time of diagnosis. Patients were clinically and/or pathologically staged and re-staged according to the latest IMIG staging system^22^.

**Table 1:** Clinicopathological characteristics of MPM patients grouped by circulating C4d levels with a cut-off of 1.5 µg/mL.

		Total (n = 55)	C4d low (n = 37)	C4d high (n = 18)	p
Gender	Male	40	28	12	0.529
	Female	15	9	6	
Age	Mean ± SD	62.0 ± 12.3	64.6 ± 11.4	56.8 ± 12.6	**0.02**
Smoker (NA = 9)	Never	15	9	6	0.700
	Former	24	17	7	
	Current	7	4	3	
Histology	Epithelioid	41	29	6	**0.002**
	Non-epithelioid	14	8	12	
IMIG Stage (NA = 11)	Early (I/II)	12	11	1	**0.035**
	Late (III/IV)	32	18	14	
Treatment (NA = 6)	CHT and/or RT	15	9	10	**0.028**
	MMT	34	24	6	
EPP	Yes	31	23	8	0.255
	No	24	14	10	
CHT (NA = 1)	Induction CHT	35	25	10	0.372
	No induction	19	11	8	

MMT, multimodal treatment; EPP, extrapleural pneumonectomy; CHT, chemotherapy; RT, radiotherapy; NA, not available

### Lack of tumor cell specific expression of C4d

**Figure 1:**
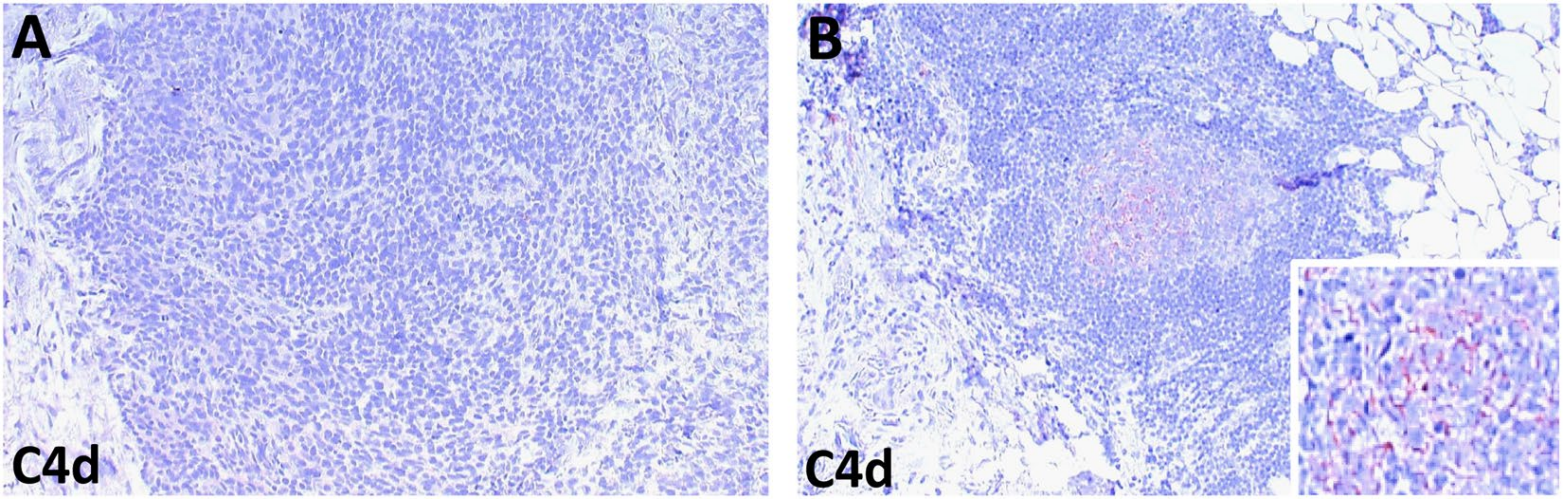
Tissue expression of C4d in MPM. **(A)** There was no C4d labeling found in MPM cells. **(B)** However, strong C4d expression was observed in the germinal center of ectopic lymphoid structures. The plasma membrane specific labeling is demonstrated in the high magnification inset.

In order to investigate C4d expression within MPM tumor tissue, we performed immunohistochemistry of 32 FFPE tissue specimens. Interestingly, we found no tumor cell specific C4d expression in these samples (Fig. 1A). Therefore, we were not able to perform any correlations between circulating C4d plasma levels and MPM tumor cell expression. As expected, several ectopic lymphoid structures of germinal centers within the tumor stroma strongly stained positive for C4d (Fig. 1B).

### Plasma levels of C4d are not increased in MPM patients

**Figure 2:**
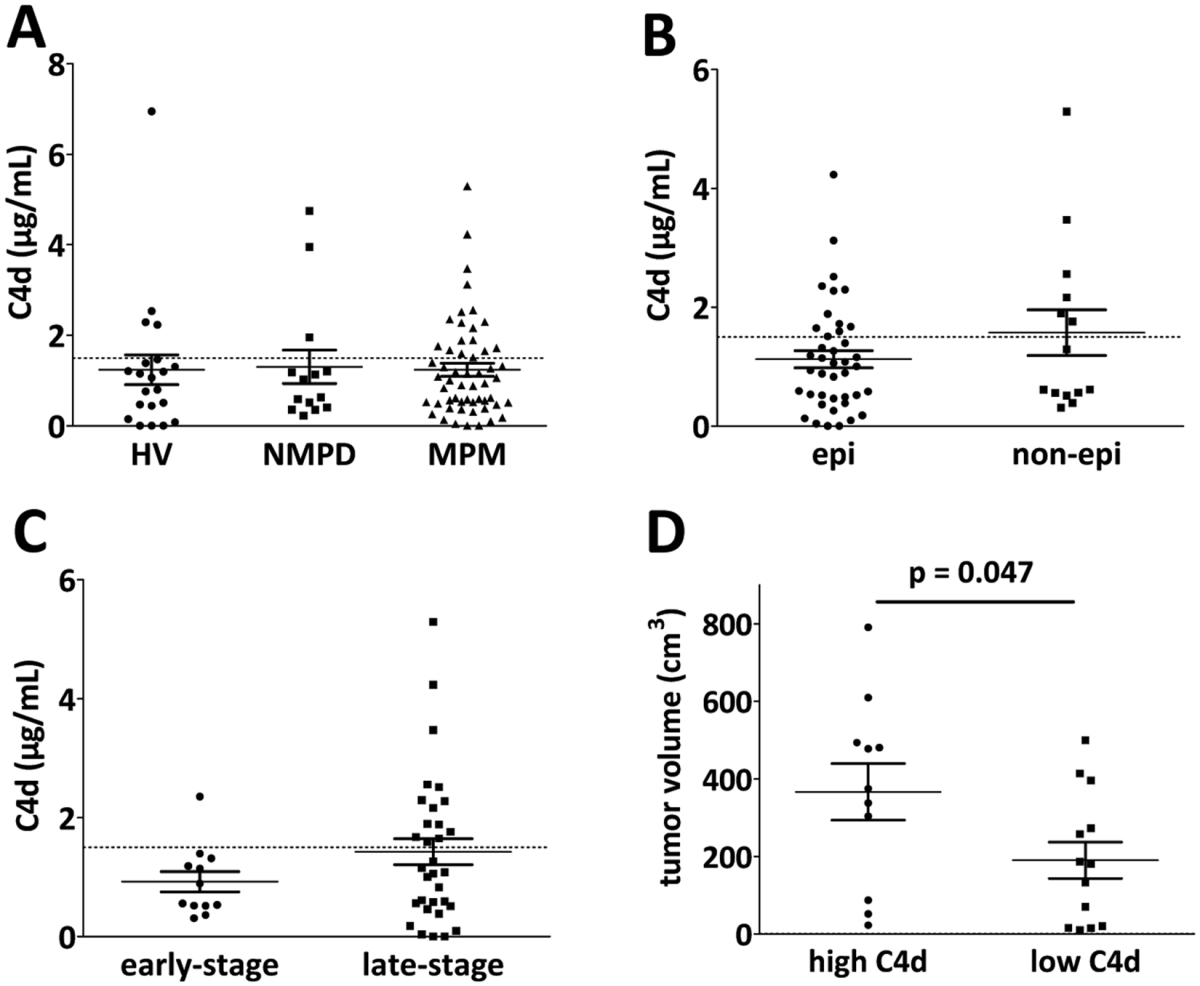
Plasma levels of C4d in MPM patients. **(A)** There were no significant differences between MPM patients and healthy volunteers (HV) or patients with non-malignant pleural disease (NMPD) in their circulating C4d levels (MPM versus HVs: Mann Whitney U test, p = 0.833; MPM versus NMPDs: Mann Whitney U test, p = 0.851). **(B)** Within the MPM cohort, we found a non-significant tendency for higher C4d levels in non-epithelioid compared to epithelioid subtype (Mann Whitney U test, p = 0.182). **(C)** Also patients with advanced disease (stage III or IV) had the tendency to have higher circulating C4d plasma levels (Mann Whitney U test, p = 0.079). **(D)** Comparison of radiologically assessed tumor volumetric data and circulating C4d levels. After dividing the cohort (cut-off 1.5 µg/mL), high C4d levels were significantly associated with a higher tumor load (366.7 ± 72.7 versus 190.3 ± 46.7 cm^3^, p = 0.047).

In order to investigate the relevance of circulating C4d as a potential diagnostic biomarker, we compared C4d plasma levels of MPM patients (n = 55), HV (n = 21) as well as of patients with NMPD (n = 14) (Fig. 2A). Interestingly, we found no significant differences between these three cohorts (MPM versus HVs: 1.24 ± 0.14 versus 1.17 ± 0.34 µg/mL, p = 0.833; MPM versus NMPDs: 1.24 ± 0.14 versus 1.30 ± 0.37 µg/mL, p = 0.851, Fig. 2A). C4d levels were much more heterogeneous in MPM patients than in HV (18 (33%) MPM patients showed a C4d level above 1.5 µg/mL - only 4 (20%) HVs had levels above 1.5 µg/mL). MPM patients presenting with epithelioid subtype tended to have lower circulating C4d levels (epithelioid versus non-epithelioid: 1.12 ± 0.14 versus 1.57 ± 0.39 µg/mL, p = 0.182, Fig. 2B). Moreover, patients presenting with advanced disease showed a tendency to have higher circulating C4d plasma levels when compared to patients with early-stage disease (IMIG stage I or II versus III or IV: 0.92 ± 0.17 versus 1.43 ± 0.22 µg/mL, p = 0.079, Fig. 2C). Interestingly, nearly all HV, NMPD as well as the majority of MPM patients had circulating C4d levels equal to or lower to our established cut-off of 1.5 µg/mL. Only a distinct subgroup of MPM patients had clearly increased circulating C4d plasma levels (Fig. 2A).

We also evaluated C4d values in terms of other main clinicopathological parameters including gender, age, smoking status and treatment regimen. Patients with epithelioid subtype, IMIG stage I/II, advanced age or those who underwent multimodality treatment were significantly more likely to have C4d levels below 1.5 µg/mL (Table 1). Notably, circulating C4d levels decreased significantly with advanced age, though with a weak correlation (Pearson r: −0.34; p = 0.003, data not shown).

### Correlation with radiologically assessed tumor volume

Since circulating C4d levels correlated with stage of disease, we evaluated whether tumor load assessed by tumor volumetry correlates with C4d levels. Pre- and post-chemotherapeutic volumetric data was available for 20 patients. Data of one patient was excluded since time between blood collection and chest CT imaging exceeded two months. Among the other patients, the average period between CT and blood collection was 21.5 days. After dichotomizing the sub-cohort according to our established C4d cut-off (1.5 µg/mL), high circulating C4d levels were significantly associated with a higher tumor load (high C4d versus low C4d: 366.7 ± 72.7 cm^3^ versus 190.3 ± 46.7 cm^3^, p = 0.047, Fig. 2D). When we directly correlated tumor volumetry with circulating C4d plasma levels, we found a non-significant tendency that C4d increased with increasing tumor volume (Pearson r: 0.34; p = 0.099, data not shown).

### Correlation with circulating inflammatory-based markers

**Figure 3:**
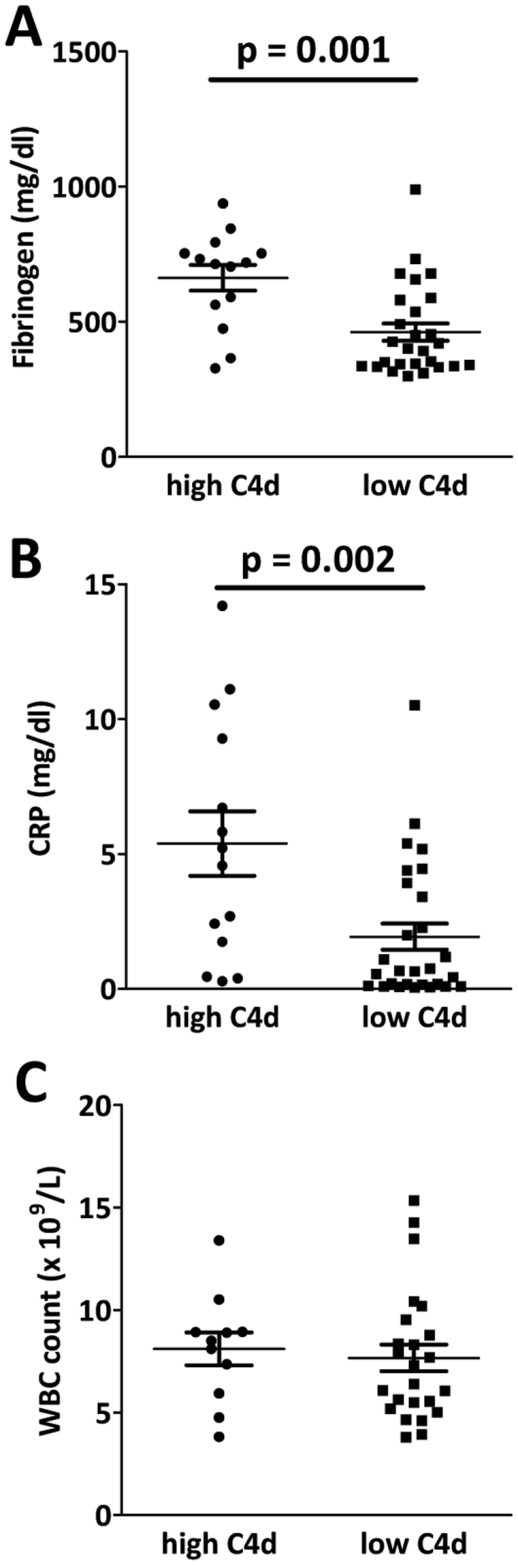
Correlations of circulating C4d levels with other inflammatory-related biomarkers. Strong correlations were found between circulating fibrinogen **(A)** as well as C-reactive protein (CRP) **(B)** and circulating C4d levels in MPM patients (Fibrinogen: Pearson r: 0.48; p = 0.001; CRP: Pearson r: 0.44; p = 0.002;). **(C)** In contrast, white blood cell count (WBC) did not correlate with circulating C4d plasma level (Pearson r: −0.14; p = 0.697).

Based on the C4d expression observed in ectopic germinal lymphoid structures within the tumor stroma, we were interested if C4d plasma levels correlate with various inflammation-related biomarkers. Higher circulating C4d levels were associated with higher plasma levels of fibrinogen (Pearson r: 0.48, p = 0.002; Mann-Whitney U test with dichotomized C4d, p = 0.001; Fig. 3A) and C reactive protein (CRP) (Pearson r: 0.44, p = 0.004; Mann-Whitney U test with dichotomized C4d, p = 0.002; Fig. 3B). We detected no correlation between C4d and white blood count (WBC) (Pearson r: −0.14, p = 0.41; Mann-Whitney U test with dichotomized C4d, p = 0.697; Fig. 3C).

### Plasma levels of C4d predict chemotherapeutic response after induction treatment

**Figure 4:**
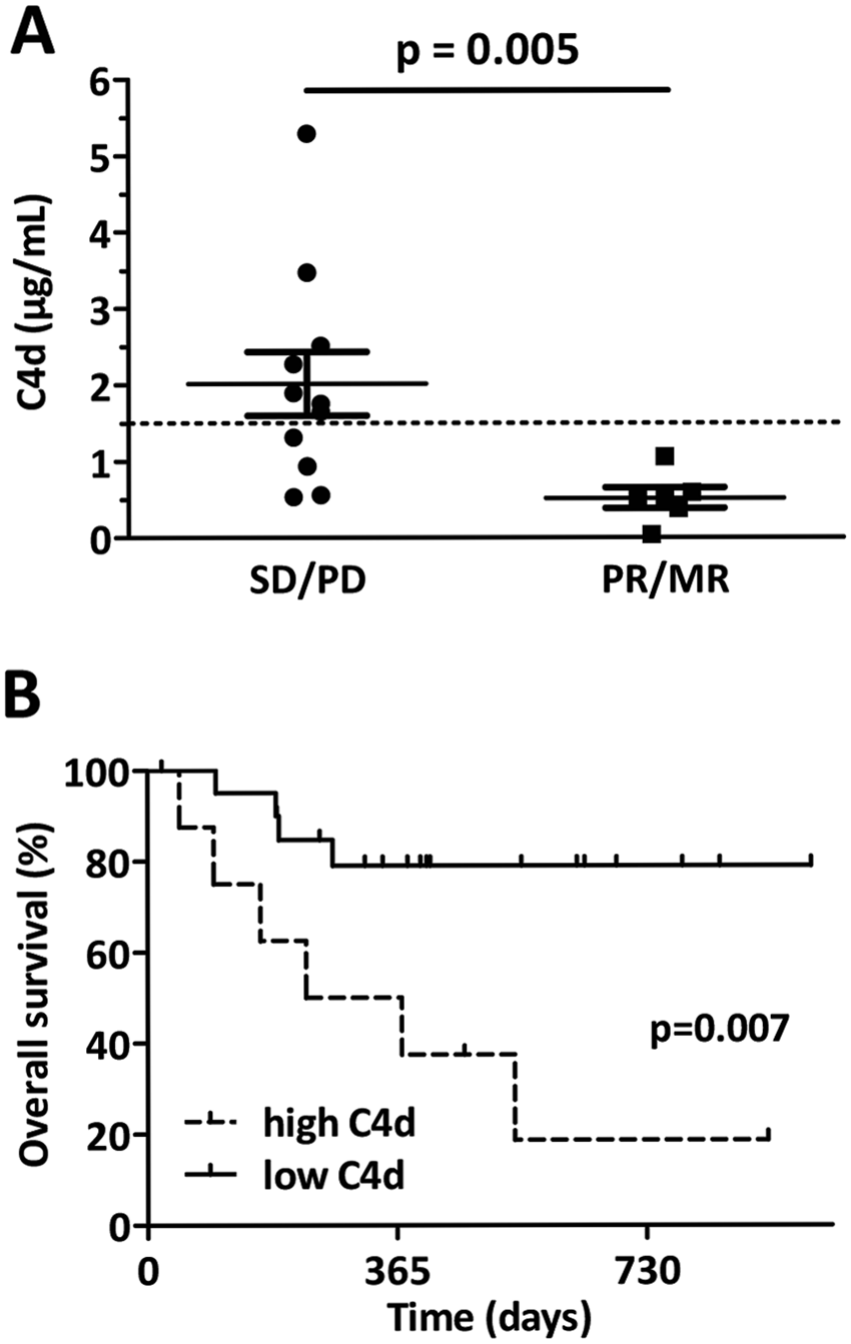
Circulating plasma level of C4d correlates with chemotherapy response and prognosis in MPM patients. (**A)** Following induction chemotherapy, patients with partial or major response (PR/MR) had significantly lower circulating C4d values when comparing to patients with stable or progressive disease (SD/PD) (0.52 ± 0.13 versus 2.02 ± 0.42 µg/mL, p = 0.005). **(B)** Kaplan-Meier survival curve of all MPM patients with plasma samples collected at time of diagnosis (n = 30). After dividing the cohort (cut-off 1.5 µg/mL) into low (n = 21) and high (n = 9) C4d plasma levels, those with low plasma C4d had significantly shorter OS (HR 7.33, CI 1.71–31.44, p = 0.007).

In order to investigate the effect of chemotherapy on circulating C4d levels, we evaluated C4d values of patients following platinum-based neo-adjuvant chemotherapy. Interestingly, patients with stable disease or progressive disease (SD/PD) after chemotherapy had significantly higher C4d levels compared to those with a partial or major response (PR/MR) (SD/PD versus PR/MR: 2.02 ± 0.42 versus 0.52 ± 0.13 µg/mL, p = 0.005, Fig. 4A). In contrary, inflammatory-based markers including fibrinogen and CRP showed no significant correlation with chemotherapeutic response (data not shown).

In a series of 12 patients we were able to measure C4d values before and after induction chemotherapy. Interestingly, patients with SD/PD showed a non-significant tendency for an increase in their circulating C4d levels following chemotherapy whereas patients with PR/MR showed a weak tendency for a decrease (relative differences between pre- and post-chemotherapy C4d levels: SD/PD + 0.39 ± 0.37 versus PR/MR −0.10 ± 0.21 µg/mL, p = 0.300, data not shown).

### Plasma levels of C4d have prognostic impact in MPM patients

To investigate the prognostic relevance of circulating plasma C4d level, we dichotomized our cohort into patients with high C4d levels (≥1.5 µg/mL; n = 9) and low C4d levels (<1.5 µg/mL; n = 21) at time of diagnosis. Patients with high plasma levels of C4d had significantly worse prognosis when compared to patients with low plasma levels (HR 7.33, CI 1.71–31.44, p = 0.007, Fig. 4B). Importantly, a multivariate cox regression analysis revealed that plasma C4d levels at diagnosis (low vs. high; HR 0.263, 95% CI 0.096–0.725, p = 0.01) influenced overall survival independently from histological subtype (epithelioid vs. non-epithelioid; HR 0.695, 95% CI 0.25–1.935, p = 0.486), IMIG stage (early vs. late; HR 0.711, 95% CI 0.176–2.875, p = 0.633) and type of treatment (multimodality treatment including radical surgery vs. other; HR 0.277, 95% CI 0.101–0.761, p = 0.013, Table 2).

**Table 2:** Multivariate Cox-regression analyses adjusted for clinical factors influencing overall survival of MPM patients.

		HR	95% CI	p
Histology	Epithelioid	0.695	0.250–1.935	0.486
	Non-epithelioid	1		
IMIG Stage	Early (I/II)	0.711	0.176–2.875	0.633
	Late (III/IV)	1		
Treatment	MMT	0.277	0.101–0.761	**0.013**
	CHT and/or RT	1		
Plasma C4d levels	Low	0.263	0.096–0.725	**0.010**
	High	1		

MMT, multimodal treatment; CHT, chemotherapy; RT, radiotherapy; NA, not available; HR, hazard ratio; CI, confidence interval.

### Correlation of circulating C4d with other complement components

**Figure 5:**
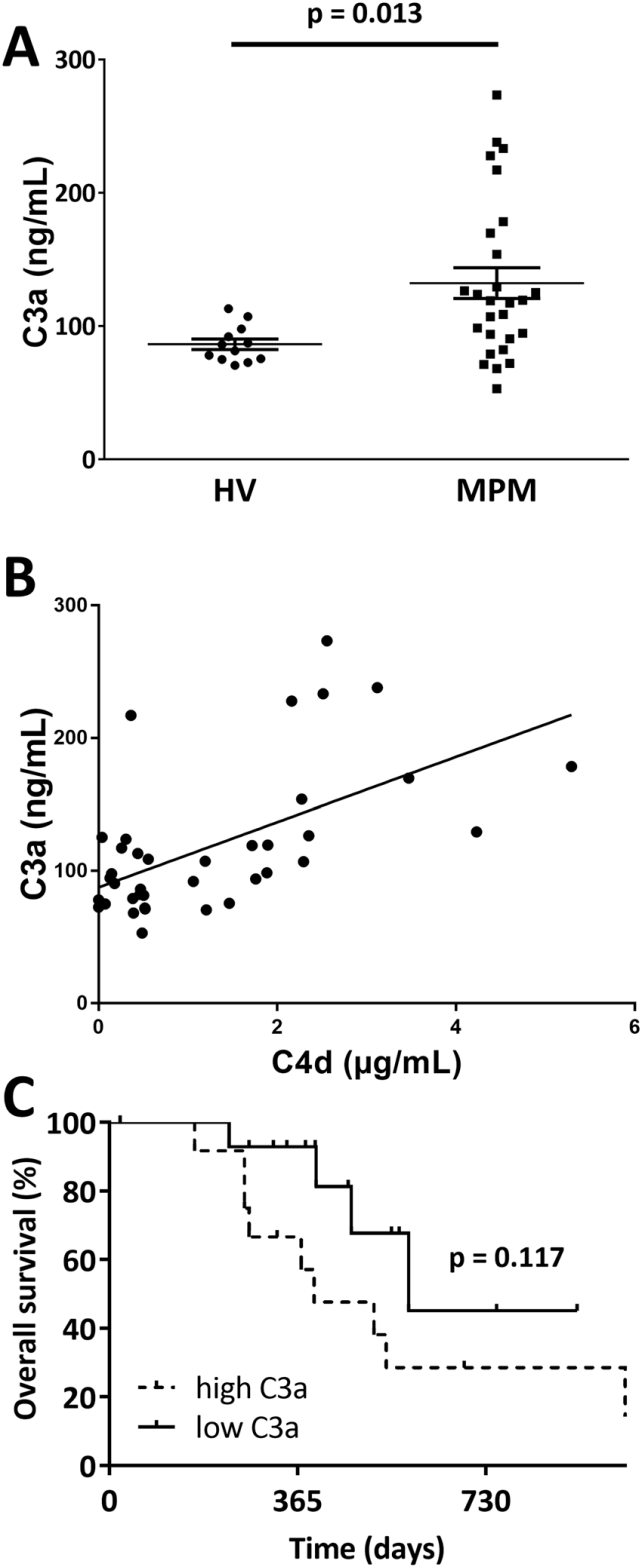
Plasma levels of C3a in MPM patients. **(A)** MPM patients had significantly higher circulating C3a levels compared to healthy volunteers (HVs) (MPM versus HVs: Mann Whitney U test, p = 0.013). **(B)** There was a modest but significantly positive correlation between circulating C3a and C4d levels (Pearson r = 0.57, p < 0.001). **(C)** Kaplan-Meier survival curve of selected MPM patients with C3a plasma samples. After dividing the cohort (median plasma C3a as cut-off: 119 ng/mL) into sub-cohorts of patients with low (n = 14) versus high (n = 13) C3a plasma levels, there was a tendency for difference in OS between the two groups (C3a plasma levels > 119ng/mL: HR 2.52, CI 0.75 – 8.38, p = 0.117).

C4d is a product of the C4 activation. C4 degradation products form together with C2a the C3 convertase which cleaves C3 releasing C3a into the surrounding. In order to investigate the specificity of circulating C4d as a representative marker for complement activation, we measured plasma C3a levels in patients with high (n = 14) and low (n = 13) circulating C4d levels as well as in HVs (n = 12). In our cohort, plasma C3a levels were significantly higher in MPM patients compared to HVs (MPM versus HVs: 132.30 ± 11.47 versus 86.45 ± 3.98 ng/mL, p = 0.013, Fig. 5A). Within the MPM cohort, C3a levels did not correlate with histological subtype (epithelioid versus non-epithelioid: 122.70 ± 13.01 versus 151.50 ± 22.24 µg/mL, p = 0.244, data not shown), stage (IMIG stage I or II versus III or IV: 119.90 ± 21.82 versus 144.10 ± 15.69 µg/mL, p = 0.406, data not shown) or age (Pearson r: −0.05; p = 0.807, data not shown). However, we found a modest but significant positive correlation between circulating C3a and C4d plasma levels (Pearson r: 0.57; p < 0.001, Fig. 5B).

Finally, in order to investigate whether C3a shows a similar reliability as a prognostic marker in MPM as C4d, we divided the selected C3a sub-cohort into low (n = 14) and high (n = 13) C3a categories by using the median plasma C3a level as cut-off (119 ng/mL). There was a tendency for decreased OS in patients with increased circulating C3a (C3a plasma levels >119 ng/mL: HR 2.52, CI 0.75–8.38, p = 0.117, Fig. 5C). Of note, a similar tendency for worse OS in patients with high circulating C4d levels was found in the same subcohort (HR 2.542, CI 0.86–7.56, p = 0.106, data not shown).

**Figure 6:**
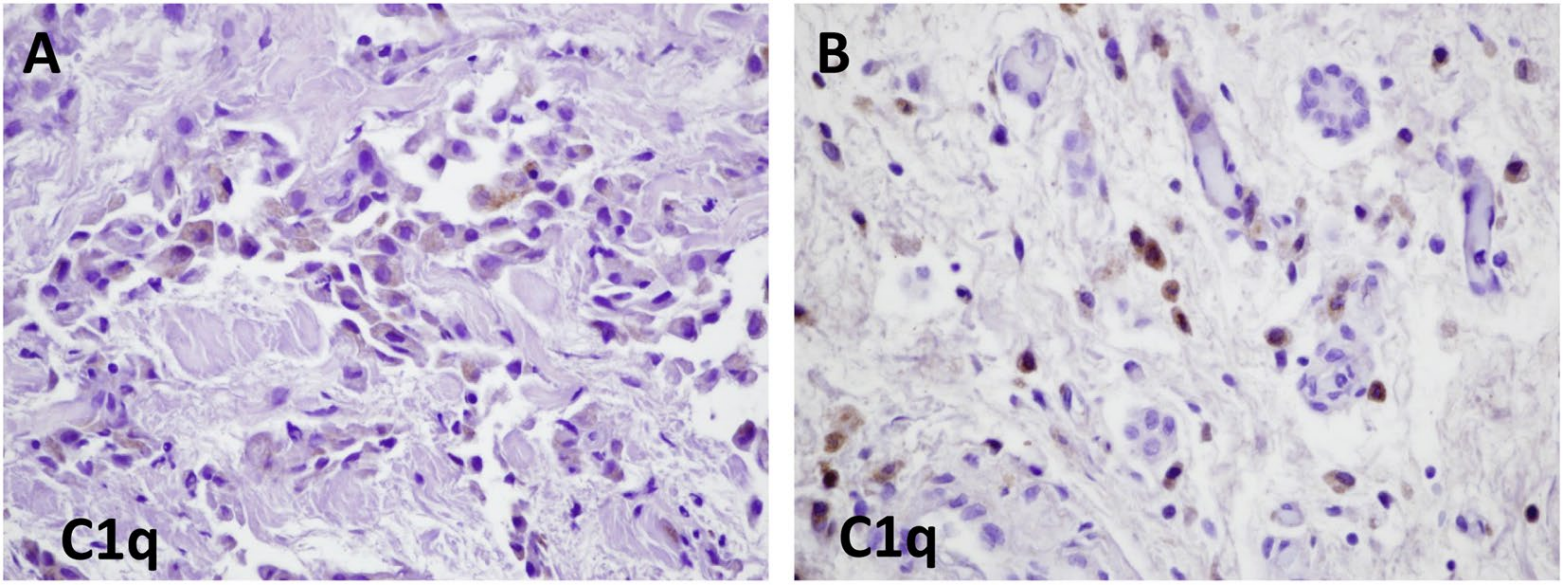
Tissue expression of C1q in MPM. **(A)** Slightly positive tumor cells were only found in two patients (both with low circulating C4d levels). **(B)** Scattered positive staining (i.e.. inflammatory cells) for C1q was found in all cases.

C4 can be activated in the classical complement pathway by C1q or in the lectin pathway by mannose binding lectin (MBL). The great majority of the resulting proteolytic cleavage product C4d remains at the side of formation and only a part is released as free C4d. As described above, C4d deposition was found in tumor samples not on cancer cells but on immune cells. To estimate whether the circulating C4d levels might be associated with the degree of tumor immune infiltration we selected 14 FFPE tissue specimens deriving from patients with high (n = 7) and low (n = 7) circulating C4d levels. Semiquantitative evaluation for tumor-associated inflammatory cells on hematoxylin-eosin stained sections revealed no correlation between circulating C4d levels and inflammatory cell infiltration (data not shown). C1q immunohistochemistry of the aforementioned 14 specimens revealed that tumors were negative for C1q in almost all samples (only two MPM samples, both deriving from patients with low circulating C4d levels, showed tumor cell-specific C1q staining; Fig. 6A). Inflammatory cells stained positive for C1q but the staining intensity was independent of circulating C4d values (Fig. 6B). Taken together these data strongly suggest that the elevated C4d levels found in late stage MPM are associated with a downstream activation of the complement cascade.

## Discussion

There is emerging evidence for the functional role of proteins of the complement cascade in tumor progression in different types of cancer^6,8–13,16,17,23^. To the best of our knowledge, this is the first study indicating the prognostic relevance of plasma C4d in a cohort of MPM patients.

Of note, MPM patients had lower C4d plasma levels compared to the lung adenocarcinoma patients from previous studies and thus we used 1.5 µg/mL as cut-off in our cohort in contrary to the 3.0 µg/mL used by others. This difference might be related to the fact that lung cancer cells have the ability to directly activate the classical complement pathway via C1q binding^12^, whereas in MPM the potential mechanisms of complement cascade activation are not investigated. In comparison to other circulating biomarkers in thoracic malignancies, circulating C4d levels show similar prognostic power for OS^24–29^. However, in contrast to the other circulating markers studied in MPM^26^, C4d levels did not differ significantly between patients and healthy controls and thus did not show diagnostic relevance.

In contrast to lung adenocarcinoma, we could not detect C4d positivity in the MPM cells by immunohistochemistry. Of note, C4d expression in tumor cells was found to correlate with prognosis, disease-stage and/or nodal invasion in certain but not all malignancies^12,13,16,17^. For instance, immunohistochemical staining for C4d in lymphomas also did not reveal tumor specific expression^23^. The lack of C4d expression/deposition in MPM cells might suggest that the complement cascade does not get directly activated by malignant cells. Importantly, we found C4d positivity in the germinal centers of ectopic lymphoid structures within the tumor stroma. Accordingly, as presented in our study, circulating C4d levels also strongly correlated with other systemic inflammatory-based markers including CRP and fibrinogen.

As C4 can be activated in the classical complement pathway by C1q, we analyzed the staining pattern of C1q in MPM tumor specimens and found modest tumor-specific C1q expression or deposition in only two patients out of 14 cases studied. This data is in line with a previous study on transcriptome analyses of MPM cell lines which demonstrated the up-regulation of C1q-binding protein (a negative regulator of C1q) in comparison to normal mesothelial cell lines^30^. However, tumor-infiltrating immune cells exhibited clear C1q positivity. Whether infiltrating immune cells themselves express the C1q or the positivity is a result of activation induced C1q binding remains unclear. Of note, our current studies on tumor tissue samples from MPM patients cannot uncover the upstream events in the complement activation.

As observed in lung adenocarcinoma^12^, our study demonstrates that circulating C4d levels correlate with disease stage in MPM. Tumor tissue specific expression of C4d was shown to correlate with disease stage and tumor size in oral squamous cell carcinoma and astrocytoma^13,17^. However, no correlation analyses between local tumor load and circulating C4d levels were performed in any of these studies. Accordingly, our study is the first to show a correlation between circulating C4d levels and radiologically assessed primary tumor volume. Our results suggest that a more advanced disease as well as a higher local tumor load might lead to amplification in complement activation that might contribute to the opsonisation of malignant cells. We further hypothesize that not the MPM tumor cells themselves but - at least in part - the local immune infiltrative cells are responsible for triggering the classical complement activation pathway. Increased tumor load may lead to an increase in the absolute immune cell infiltration resulting ultimately in higher circulating C4d levels in advanced stages of MPM. However, additional factors might also contribute to the increased circulating C4d levels observed in advanced disease. Data deriving from lupus and atherosclerosis patients showed that complement activation can be triggered by circulating apoptotic and necrotic cells, recognized by complement initiators like C1q or CRP^31–33^. Therefore, increased numbers of circulating tumor cells or amount of tumor cell debris in advanced-stage patients possibly trigger the complement cascade. This assumption could explain the observed correlation between tumor burden and systemic levels of the complement-related degradation product C4d.

In order to study the specificity of circulating C4d as a marker for complement activation in MPM patients, we additionally measured plasma C3a levels. Similar to C4d, C3a is a complement-specific cleavage product that increases following complement activation. It can be induced by the classical, the lectin or the alternative complement activation pathway and is, accordingly, considered to be an unspecific surrogate parameter for complement activation. Plasma C3a levels were increased in MPM patients in comparison to HVs and there was a modest correlation between C3a and C4d levels. The prognostic power of C3a levels showed a similar tendency as C4d within the subcohort where both components could be measured. These findings further support the presence of complement activation in certain MPM patients and underline the clinical significance of complement activation in MPM. It is important to note that while the investigation of the additional markers C3a and C1q contributes to establishing the specificity of C4d as a biomarker, these correlative studies cannot dissect the upstream events and thus the specific mechanisms of complement activation in MPM. Further studies, including preclinical *in vitro* and *in vivo* experiments, are needed to identify the specific complement activation pathways that contribute to the progression of MPM.

Similar to C4d, other circulating biomarkers in MPM like mesothelin and activin A showed similar correlations with tumor load and stage of disease^26,34–36^. To date, there exists no circulating biomarker in order to differentiate between histological MPM subtypes^35^. In our study, there was only a slight tendency for patients with non-epithelioid subtype to have higher C4d plasma values when compared to patients with epithelioid histology.

The majority of biomarker studies have focused on sample collections at the time of diagnosis and/or before surgery. In our study, we had the opportunity to analyse circulating C4d values in terms of individual chemotherapeutic response following induction chemotherapy. Remarkably, patients with progression after chemotherapy had significantly elevated circulating C4d levels when compared to those with a response following induction chemotherapy. Thus, measuring C4d levels may support the process of re-staging and re-evaluating of patients following induction chemotherapy. This is of importance, since MPM has a tendency to grow as a “rind” on the pleural surface and evaluating response and tumor expansion by RECIST criteria remains challenging^18,37^. So far our study is the first showing circulating C4d levels to directly correlate with primary tumor load and chemotherapeutic response. Therefore, additionally to conventional response assessment - measuring C4d levels might facilitate the process of re-staging in order to estimate individual treatment efficacy more comprehensively.
